# Topological arrangement of coronal segments in human callosal fibers *in vivo* tractography

**DOI:** 10.3389/fnana.2023.1097247

**Published:** 2023-05-31

**Authors:** Hui Zhang, Yuan Feng, Weiguang Li, Xili Liang, Guanglong Huang, Songtao Qi

**Affiliations:** ^1^Department of Neurosurgery, Nanfang Hospital, Southern Medical University, Guangzhou, China; ^2^Sleep Medicine Center, Department of Psychiatric, Nanfang Hospital, Southern Medical University, Guangzhou, China; ^3^Guangdong Provincial Key Laboratory of Proteomics, School of Basic Medical Sciences, Southern Medical University, Guangzhou, China; ^4^Department of Neurology, The First Clinical Medical School, Southern Medical University, Guangzhou, China

**Keywords:** corpus callosum, coronal segments, heterotopic connections, topography, tractography

## Abstract

The topography of human callosal fibers in the midsagittal corpus callosum (mid-CC), in terms of cortical termination, is inconsistent in the literature. Despite being a high-profile and controversial topic, heterotopic callosal bundles (HeCBs) have not been studied from a whole-brain perspective. Here, we used multi-modal magnetic resonance imaging data from Human Connectome Project Development to explore these two topographic aspects by combining whole-brain tractography based on multi-shell multi-tissue constrained spherical deconvolution, the post-tractography reducing-false-positive-streamline algorithm of Convex Optimization Modeling for Microstructure Informed Tractography 2, and the new cortex parcellation atlas of Human Connectome Project multi-modal parcellation, version 1.0. We proposed that the callosal streamlines would demonstrate a topological arrangement of coronal segments arranged from anterior to posterior, with each perpendicular to the long axis of the mid-CC following its natural curvature, and the adjacent segments overlapping one another owing to the existence of HeCBs. We found that the cortices connected by the coronal segments, from anterior to posterior, corresponded exactly to the cortices from anterior to posterior in the flattened cortical surfaces of this atlas, indicating the original relative positions of the neocortex before curling and flipping during brain evolution. For each cortical area defined by this atlas, the sum strength of the HeCBs was far greater than that of the homotopic callosal bundle. Our findings on the topography of the whole CC would help in further understanding the network between the bilateral hemispheres and preventing disconnection syndromes in clinical settings.

## 1. Introduction

The human corpus callosum (CC) is a white matter structure responsible for connecting the bilateral hemispheres; therefore, understanding the topography of callosal fibers is critical in clinical neuroscience. Because the CC on the midsagittal slice (mid-CC) has no distinct landmarks, Witelson (1989) partitioned it into seven parts based on its maximal straight length. In this type of partition, one recent diffusion tractography study showed that the callosal temporal, parietal, and occipital streamlines overlap, and therefore, cannot be divided by vertical lines (Hofer and Frahm, 2006). Furthermore, the use of different schemes for classifying callosal streamlines (Hofer and Frahm, 2006; Park et al., 2008; Chao et al., 2009) might be one of the reasons for the various results in the topography of human callosal fibers in the mid-CC.

Additionally, most previous studies have reconstructed callosal streamlines using Diffusion Weighted Imaging (DTI), which has a limited capacity to resolve the intersection and interdigitation between callosal fibers and association fibers (Tuch et al., 2002). These DTI studies have predominantly reconstructed streamlines targeting the medial and dorsal cortices (Hofer and Frahm, 2006), ignoring callosal temporal fibers, which pass through the posterior part of the mid-CC (Witelson, 1989). Two recent studies have used high angular resolution diffusion imaging (HARDI) to delineate callosal fibers (Chao et al., 2009; Jarbo et al., 2012). However, they were not consistent with the reconstruction of callosal temporal streamlines.

Callosal fibers include homotopic and heterotopic fibers that connect the homotopic and heterotopic regions of the bilateral cortex, respectively. The difference between these two kinds of fibers in connection quantity and spatial distribution is a high-profile and controversial theme in callosal fiber topography (Jarbo et al., 2012; De Benedictis et al., 2016). The CC was once thought to consist mainly of homotopic fibers (Tomasch, 1954). However, a recent local connectivity study found heterotopic connections to the frontal cortex in humans (De Benedictis et al., 2016; Baker et al., 2018) and premotor cortex in non-human primates (Boussaoud et al., 2005; Lanz et al., 2017). One tractography study indicated that within each of the human frontal, occipital, and parietal lobes, the average percentage of subjects with statistically significant homotopic connections across the subregions of each lobe was greater than that of those with heterotopic connections. However, the sum of statistically significant heterotopic connections at the subject or group level is far greater than that of homotopic connections (Jarbo et al., 2012). In a human case using the Nauta method for axons with anterograde degeneration, Di Virgilio and Clarke (1997) reported sparse, distant heterotopic callosal connections between the right inferior temporal cortex and Broca’s area. Each human cortical “unit” is supposed to connect with the homotopic and heterotopic “units” of the contralateral hemisphere (Innocenti et al., 2022). As the homotopy of the bilateral cortex is relative, a more precise division of the cerebral gyri might help provide new understanding to callosal fiber topography.

Therefore, in the present study, we analyzed HARDI data from Human Connectome Project (HCP) Development using advanced whole-brain tractography based on multi-shell multi-tissue constrained spherical deconvolution (Tournier et al., 2019), the post-tractography reducing-false-positive-streamlines algorithm of the Convex Optimization Modeling for Microstructure Informed Tractography (COMMIT2) (Schiavi et al., 2020), and meticulous segmentation of the cortex of the HCP multi-modal parcellation, version 1.0 (HCP_MMP1.0) (Glasser et al., 2016). We believe that the result might reshape our understanding of these two topographical aspects of homotopic and heterotopic callosal fibers in terms of their trajectory in the mid-CC and connection quantity.

## 2. Materials and methods

### 2.1. Dataset

We used publicly available preprocessed T1 and unprocessed HARDI data from the 58 oldest, healthy subjects from HCP Development.^1^ The mean (±SD) age at the time of imaging was 255.5 (±4.7) months (range: 248.0–263.0). Additionally, 36 participants were females. The handedness score according to the Edinburgh Handedness Inventory was −90, −65, −62, and −45 for one individual each, while the remaining subjects had scores >0. The spatial resolution of the 3T T1 and HARDI data was 0.8 and 1.5 mm isotropic, respectively. The *b*-values were 1,500 and 3,000 s/mm^2^ in the 93 and 92 directions, respectively, and an additional 14 interleaved *b* = 0 s/mm^2^ volumes. No experimental activity involving human subjects was conducted at the authors’ home institution. We obtained access to restricted data in the HCP. The preprocessing of HARDI data was performed using HCP pipelines (version 3.26.0) (Glasser et al., 2013) and is described in detail in our previous work (Zhang et al., 2020). T1-weighted images were linearly registered to diffusion space using the “flirt” tool in FSL 6.0.1 with 12 degrees of freedom (Jenkinson et al., 2002).

### 2.2. Quantifing callosal bundles for each subject with whole-brain tractography and COMMIT2

**FIGURE 1:**
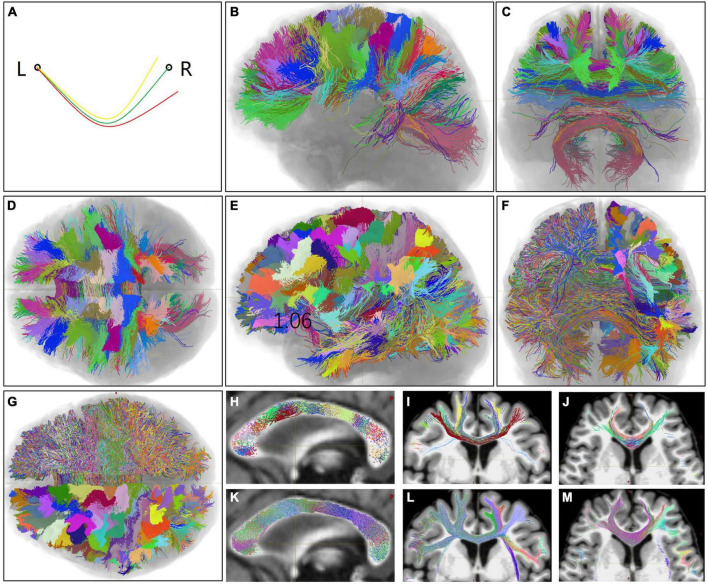
Homotopic callosal bundles (HoCBs), heterologous callosal bundles (HeCBs), and heterotopic callosal fasciculus (HCFs) of one representative subject in terms of the areas in the left hemisphere (LH). **(A)** A schematic diagram of three callosal bundles: one HoCB (green line) connects one LH area (left circle) and the homo-area in the right hemisphere (RH) (right circle), and two HeCBs (yellow and red lines) connect the LH area and two different hetero-areas in the RH. The HCF of the LH area was defined as a super bundle including all HeCBs converging in this area. Similarly, all callosal bundles can be grouped into HoCBs and HCFs for RH areas. **(B–D,H–J)** Show all HoCBs superimposed on a T1-weighted MR image, labeled in randomized colors based on the LH areas. **(E–G,K–M)** Show the HCFs of all LH areas as the HoCBs are shown. **(B–G)** Show 3D volume rendering views from the left, front, and below, respectively. **(H–M)** Show a 3D slice view of the midsagittal, coronal, and axis sections, respectively. L, left; R, right.

The fiber orientation distribution was generated, which was described in detail in our previous work (Zhang et al., 2020). Then, whole-brain tractography was performed with Mrtrix3 in the native space using the following parameters: -act, -crop_at_gmwmi, -maxlength 200, -select 5M, -cutoff 0.1 -seed_gmwmi, -angle 30, and -iFOD2 (Tournier et al., 2019). COMMIT2 was applied to the whole-brain tractogram to assign an intra-axonal signal fraction to each streamline (Schiavi et al., 2020, 2021). HCP_MMP1.0 was used to parcellate the cerebral cortex of each hemisphere into 180 areas, which further were grouped into 22 Sections. To show the topography of callosal bundles specifically in the network node file, the area in each of the 22 sections of the atlas were reordered roughly following their locations in the flattened cortical surfaces with the more inferior and posterior area listed closer to the front. Weighted callosal tractogram was obtained from weighted whole-brain tractogram by “tckedit” built in Mrtrix3 with a region of interest (ROI) in the mid-CC. Using “tck2connectome” with the parameter of “-assignment_end_voxels,” weighted streamlines of the callosal tractogram were grouped into callosal bundles by assigning each streamline to one left hemisphere (LH) area and one right hemisphere (RH) area of the node file. Callosal bundles were classified as the homotopic callosal bundles (HoCBs) or heterotopic callosal bundles (HeCBs) based on their cortical terminations at both ends defined by HCP_MMP1.0. The HoCB included streamlines connecting one LH area and the RH homotopic area and the HeCB included streamlines connecting one LH area and the RH heterotopic area. The heterotopic callosal fasciculus (HCF) of one LH/RH area, a super-bundle, consisted of all HeCBs converging in this LH/RH area (Figure 1A). Therefore, the HCF of one LH area and the HCF of the RH homotopic area are not one thing and we refered them a pair of homotopic HCFs.

### 2.3. Statistical analysis

The weighted group voxel map of the HoCB of each LH area or the HCF of each LH/RH area was obtained, in which the voxel in the mid-CC with the maximum value indicated through which streamlines of this HoCB/HCF most often crossed at the group level. A *t*-test or Wilcoxon signed-rank test for two related samples was used to explore the lateralization of each pair of homotopic HCFs or each pair of HoCB and HCF (both connecting one same LH/RH area), depending on whether the data were normally distributed. Statistical significance was set at *p* < 0.05 for all analyses. The false discovery rate of multiple comparisons was corrected by the Benjamini–Hochberg procedure, which limits alpha errors to 5% (Benjamini and Hochberg, 1995).

## 3. Results

### 3.1. Characteristics of HoCBs and HCFs in the topographical distribution at the subject level

**FIGURE 2:**
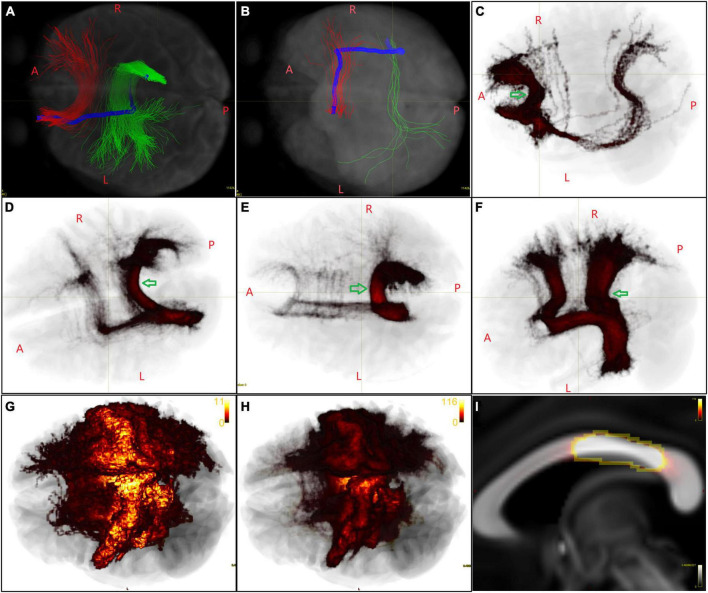
Initial callosal bundles containing false streamlines. **(A,B)** The HCF in the representative subject connecting an LH/RH area is labeled in red and green, respectively. The Type A/B streamlines (both in blue) originate from the LH/RH area, shifted some distance antero-posteriorly, turned 90°, crossed the mid-CC perpendicularly, and then reached an RH/LH area. **(C–F)** Primary group voxel maps of several HCFs of LH areas, in which Type A streamlines cross the mid-CC far from where the trunks of the HCFs cross (indicated by green arrows) and can be easily found and deleted. **(G)** In another case, taking the HCF of the left L_4 as an example, the Type A streamlines crossed the mid-CC near the trunks of the HCFs. **(H)** The image intensity range of the primary group voxel map was reset from 0 to 2× the number of subjects. **(I)** Then, voxels with the same brightness in the mid-CC were included in the ROI to delete Type A streamlines. **(G–I)** Show the same image, while in the latter two the image is shown in a same image intensity.

The initial HoCBs and HCFs of all LH areas of one representative subject (No. HCD0225529) are shown in Figures 1B–M. HoCBs mainly connected the bilateral frontal, parietal, and occipital homotopic cortices (Figures 1B–D). In addition to these cortices, HCFs densely connected the bilateral temporal and insular cortices. Even in the same cortex, the HCFs were denser than HoCBs (Figures 1E–G). The HCF connected several RH areas (Figures 2A, B) without distinguishing the cortical terminal boundaries between neighboring HCFs (Figures 1E–G). Almost all HoCBs and HCFs crossed the mid-CC perpendicularly (Figures 1H, K). The trajectories of the HoCBs in the mid-CC were organized with relatively distinct boundaries between neighboring HoCBs (Figures 1H–J), while the trajectories of the HFCs in the mid-CC had fuzzy boundaries between neighboring HFCs (Figures 1K–M).

### 3.2. Initial callosal bundles containing false streamlines

**FIGURE 3:**
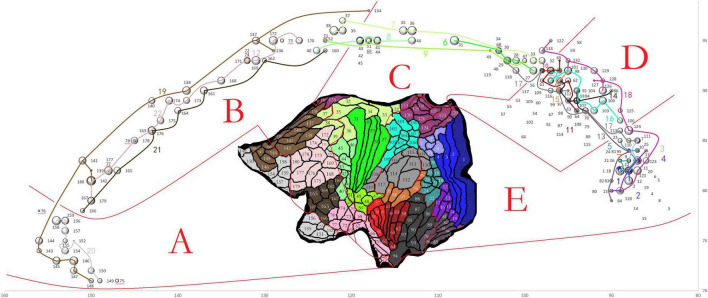
The connection strength and distribution in the mid-CC of HCFs of LH areas at the group level shown in one bubble diagram. The x and y coordinates of each bubble center represent the y and z coordinates of the voxel in the final group voxel map of the HCF of one LH area in the mid-CC with the maximum value, which indicates where the HCF frequently crosses the mid-CC at the group level. The diameter of the bubble was 10% of the log_2_ of the mean CS of the HCF at the group level. Each bubble was labeled in black with the sequence number of the stand-for node in the node file shown in the inset of the flattened cortical surfaces adapted from Glasser et al. (2016). Therefore, all bubbles were divided into five parts **(A–E)** according to the locations of their stand-for areas in the surfaces. Additionally, the bubbles, standing for the areas within one same section of the atlas, were strung together with one line labled by the sequence number of the corresponding section in the atlas. The line and the corresponding number were in the same color as that used for the cortex section in the inset. The bubbles, standing for the areas within section 12 of the atlas, could not be strung due to their dispersed distribution. They were labled by the underlines.

From the initial callosal bundles obtained from the representative subject, we found that the HCFs of many areas included a small number of streamlines that originated from LH/RH areas, shifted some distance antero-posteriorly in the ipsilateral hemisphere, turned 90°, crossed the mid-CC vertically, eventually reaching the contralateral areas. We referred to these long-span streamlines that originated from an LH/RH area as Type A and Type B streamlines (the blue streamline in Figures 2A, B), which correspond to the yellow and red bundles, respectively, shown in the second column of the top row (false-positives) of Figure 3 in the COMMIT2 paper (Schiavi et al., 2020). In Figures 2C–F, the initial group voxel maps of several LH HCF showed Type A streamlines at the group level, which were obtained with the following steps: the voxel map for each initial LH HCF of each subject was obtained using “tckmap” and then registered to FSL_HCP1065_FA_1 mm.nii.gz using the FNIRT tool in FSL 6.0.1 (Jenkinson et al., 2002) and the outputs for all subjects within the same LH HCF were added together. Therefore, we deleted these streamlines before further post-tractography analysis with the following steps: one ROI was drawn on the mid-CC in the above-mentioned template space for each LH HCF, which contained a cluster of voxels through which most streamlines, except Type A, passed, as indicated by each arrow in Figures 2C–F. Figures 2G–I shows that the image intensity range of the group voxel map of the left L_4 HCF was set from 0 to 2× the number of subjects. Then, voxels with the same brightness in the mid-CC were included in the ROI, to delete Type A streamlines that crossed the callosum near the “true” streamlines. After the Type A streamlines were deleted in the native space for all LH HCFs separately, all modified LH HCFs for each subject were merged into a new callosal tractogram for each subject. The new callosal tractogram for each subject was then divided into RH HCFs. The same method was used for deleting Type B streamlines from RH HCFs, and a final callosal tractogram was created by combining all modified RH HCFs.

### 3.3. Connection strength and topographical distribution in the mid-CC of HoCBs and HCFs at the group level

COMMIT2 was finally applied to the modified whole-brain tractogram, merged from the final callosal tractogram and whole-brain tractogram without callosal streamlines. For that the trajectories of the HFCs in the mid-CC had fuzzy boundaries between neighboring HFCs, we present a Bubble diagram (Figure 3) to simultaneously specify the connection strengths (CSs) and distributions in the mid-CC of LH HCFs in terms of their cortical termination. The x and y coordinates of the voxel in the mid-CC with the maximum value of the final group voxel map of the HCF of each LH area, with a mean weighted CS ≥ 2 were used to set the bubble’s location, and the mean weighed CS was used to set the bubble’s diameter, which was calculated as 10% of the log_2_ of the mean weighted CS. Each bubble corresponds to an LH area. Trajectories of HCFs in the mid-CC in the dorsal-ventral direction corresponded to the locations of the LH areas in the flattened cortical surfaces (The inset in Figure 3) when the trajectory locations were determined using the scheme of the callosal AP axis. All bubbles were divided into five parts according to the locations of the corresponding areas on the flattened cortical surfaces. In doing so, we attempted to show that the trajectories of HCFs in the mid-CC in the AP direction corresponded to the locations of the LH areas in the flattened cortical surfaces, when the trajectory locations were determined in the scheme of the callosal AP axis. Overall, the HCFs were arranged in the mid-CC according to the locations of their connecting areas, from anterior to posterior and from dorsal to ventral, in the flattened cortical surfaces. For example, in part A, the HCFs connecting the anterior part of section 19 crossed the mid-CC above where those of section 20 crossed, and the HCFs connecting the more anterior areas of sections 19 or 20 crossed the mid-CC before those of posterior areas of the same section.

### 3.4. Lateralization of homotopic HCFs

**FIGURE 4:**
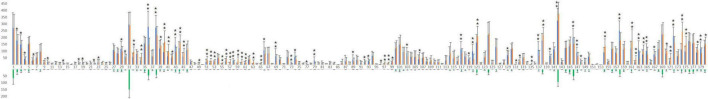
Comparisons of connection strengths (CS) between HoCBs and HCFs of each LH area, and between each pair of homotopic HCFs. From the left to right sides of the axis, 180 combinations of green bars below the axis and blue and red bars above the axis indicate the mean + SD of the CSs of the HoCBs and HCFs of 180 LH areas (arranged from Node 1 to 180 of the node file, see the inset of Figure 3), and HCFs of 180 RH areas (arranged from Node 181 to 360 of the node file), respectively. As the bars show, all HCFs of the LH/RH areas are denser than the corresponding HoCBs. The Wilcoxon signed-rank test for two related samples indicated a significant difference in the CSs of 82 pairs of homotopic HCFs whose bars are marked by * indicating *p* < 0.05 or ** indicating *p* < 0.001, with 40 pairs of homotopic HCFs dominant on the left side and 42 on the right side.

From the callosal connectome of each subject, the CSs of HoCBs of LH areas and HCFs of LH and RH areas were extracted. Using the histogram shown in Figure 4, we found that the HCFs of all LH/RH areas were denser than the corresponding HoCBs, especially in the temporal callosal bundles. Therefore, the Wilcoxon signed-rank test for two related samples was used to explore the lateralization of each pair of homotopic HCFs, which indicated that there was a significant difference in the CSs of 82 pairs of homotopic HCFs, with 40 pairs of homotopic HCFs dominant in the LH and 42 in the RH. When allocating the 82 pairs of bilateral homotopic cortical areas into 22 numbered sections, more than half of the areas in sections 2, 5, 6, 12, 14, and 17 had dominant HCFs in the LH. In contrast, more than half of the areas in sections 8, 9, 10, 11, 15, and 22 area had dominant HCFs in the RH. Bilateral symmetry was observed in other sections. For example, in Broca’s complex (Xiang et al., 2010), Brodmann 44 was right-dominant and Brodmann 45 was left-dominant. In the five areas of Brodmann 47, only p47r was left-dominant.

### 3.5. Coronal segment arrangement of callosal streamlines

**FIGURE 5:**
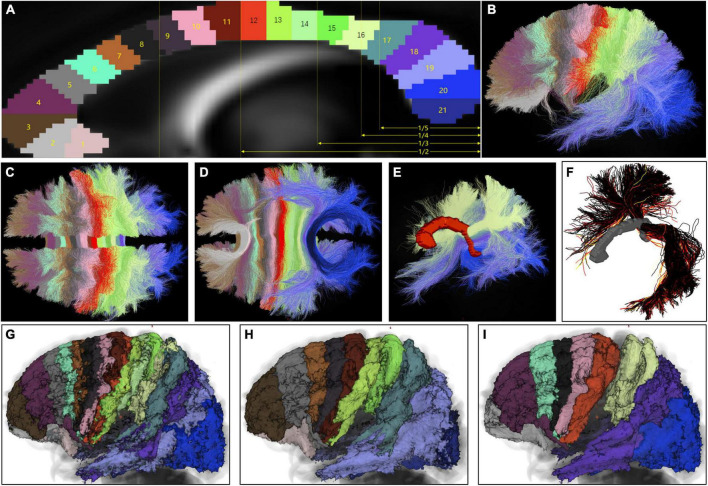
Topological arrangement of coronal segments in callosal streamlines at the subject and group level. **(A)** A total of 21 mid-CC ROIs were drawn in the standard space from the rostrum to the splenium. Most previous studies partitioned the mid-CC based on its maximal straight length. Therefore, the proportional scales of this length of the mid-CC are shown in yellow in order that our partition scheme would be compared with previous schemes. **(B–D)** With these ROIs, the weighted final callosal tractogram of the representative subject was divided into 21 sub-tractograms. **(E)** The coronal segment of sub-tractograms 16–20, which include temporal streamlines and streamline trajectories. **(F)** The weighted streamlines of the representative subject converging into the left temporal cortex, which connects part of the opposite parietal and occipital cortices, in addition to the opposite temporal cortex. **(G)** Final group voxel maps of 21 sub-tractograms superimposed on a T1-weighted MR image in the standard space, using similar colors as those of the corresponding mid-CC ROIs in panel **(A)**. **(H,I)** Final group voxel maps of odd and even numbers of sub-tractograms, respectively, shown in the same way as **(G)**. **(A,B,G–I)** Views of the left side. **(C,D)** Are viewed above and below, respectively. **(E,F)** Are left dorsolateral views.

As shown in Figure 1, from the mid-CC, callosal streamlines set out vertically to the midsagittal plane, and passed bilaterally to the bilateral cortex in the coronal plane perpendicular to the natural curvature of the part of mid-CC where these streamlines crossed. Therefore, we defined the callosal antero-posterior (AP) axis by imagining the mid-CC straightened antero-posteriorly from the rostrum to the splenium. In the callosal AP axis scheme, the point in the rostrum was anterior to the point in the genu, and the point in the originally anterior part of the genu was dorsal to the point in the originally posterior part of the genu. To determine bilateral cerebral termination of callosal streamlines in terms of their trajectories in the mid-CC, 21 mid-CC ROIs on the mid-CC of FSL_HCP1065_FA_1 mm.nii.gz were drawn manually. The long axis of each ROI was meant to be perpendicular to the natural curvature of the mid-CC, and the width of each ROI was 2–3 voxels (Figure 5A). After being registered back to the native space of each subject, these 21 ROIs were used to divide the weighted final callosal tractograms into 21 sub-tractograms. As shown in Figures 5B–E, the range of cortical termination of each sub-tractogram of the representative subject and the overlap in cortical termination between the adjacent sub-tractograms indicate that callosal streamlines limitedly spread over one imaginary coronal segment (inspired by segments of Annelida), rather than strictly in one coronal plane, both of which cross the corresponding mid-CC ROI and follow the long axis of the ROI. Even in the coronal segments of sub-tractograms 16–20, which include temporal streamlines, the trajectories of the streamlines follow the pattern described above (Figure 5E). Figure 5F shows the weighted streamlines of the representative subject converging into the left temporal cortex, which connects part of the opposite parietal and occipital cortices, in addition to the opposite temporal cortex. These streamlines were also arranged the topography of coronal segments. The coronal segment arrangement of callosal streamlines can also be seen at the group level using the final group voxel maps for the sub-tractograms (Figures 5G–I). When the mid-CC was straightened in the callosal AP axis, all callosal streamlines were almost in their coronal segments like segments of Annelida.

## 4. Discussion

In this study, we proposed that callosal streamlines demonstrate a topological structure of coronal segments perpendicular to the long axis of the mid-CC following its natural curvature, with the adjacent segments overlapping each other in their cortical termination. The arrangement of the cortical terminations of the coronal segments, from anterior to posterior, corresponded exactly to that of the cortices in the flattened cortical surfaces of the HCP-MMP 1.0 atlas. For each cortical area, the summary CS of all HeCBs was far greater than that of all HoCBs.

In hedgehogs, the entire neocortex is composed of sensory, motor, and insular areas. The insular cortex is ventral to the motor and primary somatosensory areas and anterior to the auditory cortex. With the evolutionary development of the neocortex, the prosimian galago developed two association cortices: the anterior cortex anterior to the premotor cortex and the posterior cortex posterior to the primary somatosensory cortex and primary auditory cortex and anterior to the primary visual cortex. The posterior cortex includes the posterior parietal and lateral temporal cortices, which are ventral to the parietal cortex. In humans, the temporal cortex is more developed antero-posteriorly, and the temporal pole reaches the location ventral to the anterior association cortex (Nieuwenhuys, 1994). Therefore, the flattened cortical surfaces of the HCP-MMP 1.0 atlas showed rough relative positions between the human neocortex in the primary phase. Our findings indicated that the relative positions between the trajectories in the mid-CC of human callosal streamlines connecting the frontal, insular, parietal, temporal, and occipital cortices corresponded to those between their cortical termination in the flattened cortical surfaces. Thus, although selection pressure over evolutionary time scales have resulted in various expansion rates in different human cortical areas compared to those of the hedgehog and prosimian galago, the relative positions between the primary cortices of humans before the human cortices curled and flipped because of brain evolution was conserved in the mid-CC. Moreover, our findings showed that if the mid-CC was partitioned into 21 parts whose long axes were perpendicular to the long axis of the mid-CC following its natural curvature rather than the horizontal maximal straight length used in previous studies (Witelson, 1989), each side of the neocortex would be partitioned into 21 parts, each including one strip of the neocortex with its long axis roughly following the long axis of the corresponding mid-CC ROI. Two studies used retrograde tracers to show that heterotopic callosal fibers of non-human primates target the premotor areas majorly from the opposite homotopic area with greater range (Boussaoud et al., 2005; Lanz et al., 2017). One previous study (Boussaoud et al., 2005) shows that the opposite cortical termination of callosal fibers connecting the premotor areas formed a larger region, roughly the same as the aforementioned strip. Therefore, we propose the concept of “partially overlapping coronal segments” in callosal streamlines, which can explain why the callosal temporal, parietal, and occipital streamlines cannot be divided by vertical lines (Hofer and Frahm, 2006). Furthermore, we provided a map of the cortical termination of callosal streamlines in terms of their trajectory in the mid-CC, which does not need to be memorized in detail if it is kept in mind that the cortical terminations of human callosal fibers corresponded to their trajectories in the mid-CC in the pattern of the arrangement of coronal segments.

The existence of HeCBs has been locally verified (Aboitiz et al., 2003; Clarke, 2003; Boussaoud et al., 2005; Jarbo et al., 2012; De Benedictis et al., 2016; Lanz et al., 2017; Baker et al., 2018), and the range of cortical termination in the superior-inferior direction of HeCBs has been previously studied (De Benedictis et al., 2016). As more callosal streamlines were reconstructed and smaller units of cortical area were used in this study, our result of HeCB of any area being denser than HoCB of the area was expected, as homotopy is relative (Innocenti et al., 2022). In non-human primates, heterotopic callosal connections have been reported to have two types: (i) between one part of a cortical area and one heterotopic part of the opposite homotopic cortical area, or (ii) between one part of a cortical area and one part of an opposite heterotopic cortical area near the opposite homotopic cortical area (Di Virgilio and Clarke, 1997). HoCBs and these two types of HeCbs could be interchanged if the definition of bilateral homotopic cortical areas was changed. In a previous study (Boussaoud et al., 2005), the lateral premotor cortex was divided into four regions, and the homotopic callosal fibers were slightly denser than heterotopic fibers. However, when the cortex was partitioned into seven areas, as in our study, the result differed, challenging the idea that callosal fibers are mainly homotopic (Tomasch, 1954). Some may argue that false-positive streamlines caused this difference, as they are an inevitable drawback of the existing diffusion tractography techniques. However, with COMMIT2, our analysis strategy was more advanced and rigorous than those previously employed, theoretically resulting in fewer false-positive streamlines. In addition, our reconstructed heterotopic bundles did not include distant heterotopic callosal connections, in our attempt to further remove false-positive streamlines. Therefore, our result was likely not due to an increase in false-positive streamlines. The overlap between cortical terminations of adjacent coronal segments presents a compensatory and redundant protection mechanism. That is, localized injury to the CC could be compensated for by adjacent fibers without an obvious loss of function. Moreover, different sites of callosal lesions result in various sites of hemispheric disconnection from the prefrontal to the occipital cortex, causing disconnection syndromes (Jea et al., 2008; Berlucchi, 2012). Although the anatomic and physiologic factors responsible for disorders after CC sectioning are not entirely known, neurosurgical practice has found that *a* < 2-cm incision in the CC does not result in disconnection syndromes (Asgari et al., 2003).

Because the COMMIT2 study could not completely remove the Type A and B streamlines (Schiavi et al., 2020), we deleted these streamlines before further post-tractography analysis. Although this deletion method contains arbitrary steps, it would help further decrease the number of false-positives in COMMIT2. However, no standard method has been provided to determine to what extent false-positive and false-negative problems occur. A more detailed discussion of the diffusion tractography method is beyond the scope of this study. Of note, caution should be exercised in terms of the maximal range of the contralateral cortex in the AP direction within the flattened cortical surfaces of the HCPMMP 1.0 atlas connected by one HeCB. These findings should be verified by other methods, including fiber tracing. For the first time, we proposed the laterality of pairs of homotopic HCFs and compared callosal temporal homotopic and heterotopic bundles. Further study is required for discussing the implications of these two parts of our results.

## 5. Conclusion

Our study proposes a new arrangement of coronal segments for callosal fibers in the mid-CC in terms of their cortical termination based on whole-brain tractography. Supported by evidence from anatomical studies and neurosurgical practice, these findings would help in our understanding of the network between the bilateral hemispheres and preventing disconnection syndromes in the clinical settings.

## Data availability statement

The original contributions presented in this study are included in the article/supplementary material, further inquiries can be directed to the corresponding authors.

## Ethics statement

Ethical review and approval was not required for the study on human participants in accordance with the local legislation and institutional requirements. The ethics committee waived the requirement of written informed consent for participation.

## Author contributions

HZ: conceptualization, methodology, formal analysis, resources, writing – original draft preparation, and writing – reviewing and editing. YF: data curation, writing – original draft preparation, and writing – reviewing and editing. WL: data curation, visualization, and investigation. XL: writing – original draft preparation. GH: writing – reviewing and editing, investigation, and data curation. SQ: writing – review and editing, supervision, and project administration. All authors contributed to the article and approved the submitted version.

## Footnotes

1
https://www.humanconnectome.org/study/hcp-lifespan-development/article/lifes
